# Airway ultrasound to detect subglottic secretion above endotracheal tube cuff

**DOI:** 10.1186/s13089-023-00318-5

**Published:** 2023-05-06

**Authors:** Osman Adi, Chan Pei Fong, Roslanuddin Mohd Sallehuddin, Azma Haryaty Ahmad, Kok Meng Sum, Zulrushdi Md Yusof, Gabriele Via, Guido Tavazzi

**Affiliations:** 1 Resuscitation and Emergency Critical Care Unit (RECCU), Trauma and Emergency Department, Hospital Raja Permaisuri Bainun, Ipoh, Perak Malaysia; 2 Trauma and Emergency Department, Hospital Sultanah Bahiyah, Alor Star, Kedah Malaysia; 3 Department of Anesthesiology and Intensive Care, Beacon Hospital, No. 1, Jalan 215, Off Jalan Templer, Section 51, 46050 Petaling Jaya, Selangor Malaysia; 4 Department of Radiology, Raja Permaisuri Bainun Hospital, Jalan Raja Ashman (Jalan Hospital), Ipoh, Perak Malaysia; 5 Cardiac Anesthesia and Intensive Care – Cardiocentro Ticino, Lugano, Switzerland; 6 Department of Clinical, Surgical, Diagnostic and Pediatric Sciences, University of Pavia, DEA Piano-1, Fondazione IRCCS Policlinico S. Matteo, Viale Golgi 19, 27100 Pavia, Italy; 7 Department of Anesthesia and Intensive Care Unit, Fondazione IRCCS Policlinico S. Matteo, Pavia, Italy

**Keywords:** Ventilator-associated pneumonia prevention, Microaspiration, Subglottic secretion, Point-of-care ultrasound, Focused airway ultrasound

## Abstract

**Background:**

Subglottic secretion had been proven as one of the causes of microaspiration and increased risk of ventilator-associated pneumonia (VAP). The role of ultrasound to detect subglottic secretion has not yet been established.

**Purpose:**

The purpose of this study is to determine the sensitivity and specificity of upper airway ultrasound (US) in the detection of subglottic secretions as compared to computed tomography (CT) scanning.

**Material and methods:**

A prospective observational study was carried out in adult trauma patients requiring mechanical ventilation and cervical CT scan. All patients had an endotracheal tube cuff-pressure maintained between 20 and 30 cm H_2_O. Airway US was performed at the bedside immediately before the patient was transferred to the CT scan suite. The sensitivity, specificity, and positive/negative predictive values (PPV, NPV) of the upper airway US detection of subglottic secretions were then calculated and compared with CT findings.

**Results:**

Fifty participants were consecutively included. Subglottic secretions were detected in 31 patients using upper airway US. The sensitivity and specificity of upper airway US in detecting subglottic secretion were 96.7% and 90%, respectively (PPV 93.5%, NPV 94.7%). 18 (58%) patients with subglottic secretions developed VAP during their ICU stay (*p = 0.01*). The area under the receiver operating curve (AUROC) was 0.977 (95% CI 0.936–1.00).

**Conclusions:**

Upper airway US is a useful tool for detecting subglottic secretions with high sensitivity and specificity.

**Clinical implications:**

This study shows:

1. Upper airway US may aid in detecting subglottic secretions, which are linked to VAP.
2. Detecting subglottic secretions at the bedside aids in determining the best frequency of subglottic aspiration to clean the subglottic trachea.
3. Upper airway US may also aid in detecting the correct ETT position.

*Trial registration* Clinicaltrials.gov. Clinicaltrials.gov identifier NCT04739878 Date of registration 2nd May 2021 URL of trial registry record https://clinicaltrials.gov/ct2/show/NCT04739878.

**Supplementary Information:**

The online version contains supplementary material available at 10.1186/s13089-023-00318-5.

## Key results

- Upper airway ultrasound had a sensitivity of 96.7% (29/30) and specificity of 90% (18/20) in detecting subglottic secretions in 50 mechanically ventilated adult patients. The negative predictive value (NPV) of airway ultrasound compared to CT was 94.7%, and positive predictive value (PPV) was 93.5%.
- There was a significantly higher incidence of ventilator-associated pneumonia in patients with subglottic secretions compared to those with no subglottic secretions (58% vs 11%, *p = 0.01*).

## Introduction

Orotracheal intubation is provided to many critically ill patients who requires airway protection and/or ventilator support. To maintain an appropriate airway seal, the endotracheal tube (ETT) cuff pressure should be maintained between 20 and 30 cmH_2_O as a way to minimize airway leak and avoid compromising the integrity of the tracheal mucosa [1]. The accumulation of secretions above the endotracheal tube (ETT) cuff, are not easily removed with tracheal tubes that lack subglottic secretion drainage (SSD) ports and therefore, predisposing to microaspiration and ventilator-associated pneumonia) [2–5]. Nevertheless, the SSD technique is still underutilized [4, 5]. The main reasons are costs and safety issues of SSD, which may cause prolapse of tracheal mucosa into the suction port, especially with continuous aspiration of the subglottic area [6]. Therefore, intermittent SSD is recommended, and its efficiency may be improved by synchronizing drainage with subglottic secretions accumulation [6, 7].

Subglottic secretions above the ETT cuff can be detected directly by aspiration of the subglottic area, [1] or through visualization by imaging [8]. CT features of subglottic secretions include complex shapes, internal air bubbles, location at dependent portion, and CT Hounsfield < units < 21.7 [9].

To our knowledge, studies using airway ultrasound (US) to visualize subglottic secretions in intubated patients are scarce [10, 11]. The main objective of our study is to compare the performance of US with CT in detecting subglottic secretion above the ETT tube cuff.

## Methods

### Study design, setting and ethical consideration

Consecutive trauma patients admitted to the Emergency Department of Raja Permaisuri Bainun Hospital from November 2021 to January 2022 who required endotracheal intubation and a cervical computed tomography (CT) scan were enrolled.

Ethical approval was obtained from the Medical Research and Ethics Committee of Malaysia Ministry of Health was granted [NMRR-21-1852-61475 (IIR)]. Study was also registered at the ClinicalTrials.gov (Identifier: NCT04739878). Written informed consent was obtained from the patients or their next of kin.

### Participants

The inclusion criteria were: (1) ≥  18 years old; (2) requirement of endotracheal intubation (either via mouth or nose); and (3) clinically indicated cervical CT scan.

Patients were excluded if they had any of the following criterion: (1) subcutaneous emphysema of the neck; (2) scars or surgical dressing around the neck which can lead to difficulty in obtaining optimal ultrasound images.

### Intervention

All patients were kept in the supine position, intubated with an Idealcare (Ideal Healthcare Sdn Bhd, Malaysia) oral high volume low pressure cuffed ETT, and then mechanically ventilated. The decision to intubate the patient was made by the primary care team, without the participation of the investigator team. The ventilator machine setting was initiated at the discretion of the treating physician.

The ETT pilot balloon was connected to a cube pressure tube with filter, (Promepla S.A.M, Monaco, France) and inflated with air to adequately seal the airway. The ETT cuff pressure was continuously measure, and monitored using the IntelliCuff of the Hamilton G5 ventilator (Hamilton, Switzerland). The target cuff pressure was set between 20 and 30 cm H_2_O [12]. IntelliCuff^®^ automatically adjusted the cuff pressure within these values. In the event of a damaged cuff, the device generated an alarm while simultaneously increasing the pressure as a way to maintain the desired cuff pressure.

The following data were recorded at the time of intubation: patients’ demographics, size of the ETT, and physiological parameters. Studied patient outcomes include the incidence of VAP, mortality, intensive care unit (ICU) and hospital length of stay, and days on mechanical ventilation. The CT scan was used as the gold standard for delineating supraglottic secretions. All patients were admitted to the ICU after CT scan.

### Ultrasound examination

Airway US was performed at the bedside immediately before the patient was transferred to the CT scan suite. This ensures that the subglottic secretions that was observed by US would also be detected by CT.

Airway US was performed by critical care physicians and emergency physicians who were trained in critical care sonography with a minimum of 5 years of experience. All investigators had undergone airway US training by the World Interactive Network Focused On Critical Ultrasound (WINFOCUS).

**Fig. 1:**
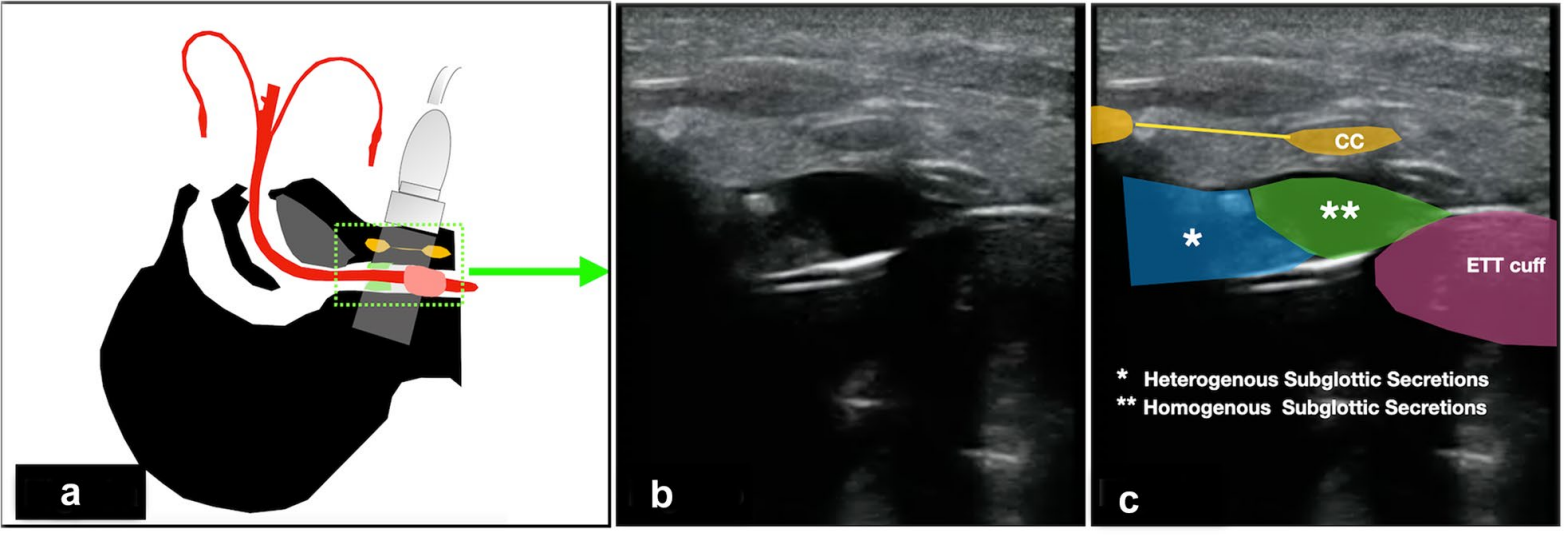
**a** A longitudinal view was obtained with the transducer placed at the anterior midline of the neck; **b** airway ultrasound (longitudinal view) showing subglottic secretions. **c** Relation of heterogenous subglottic secretions (blue watermark) and homogenous subglottic secretions (green watermark) with endotracheal (ETT) cuff, cricoid cartilage (CC) and cricothyroid membrane (yellow line)

**Fig. 2:**
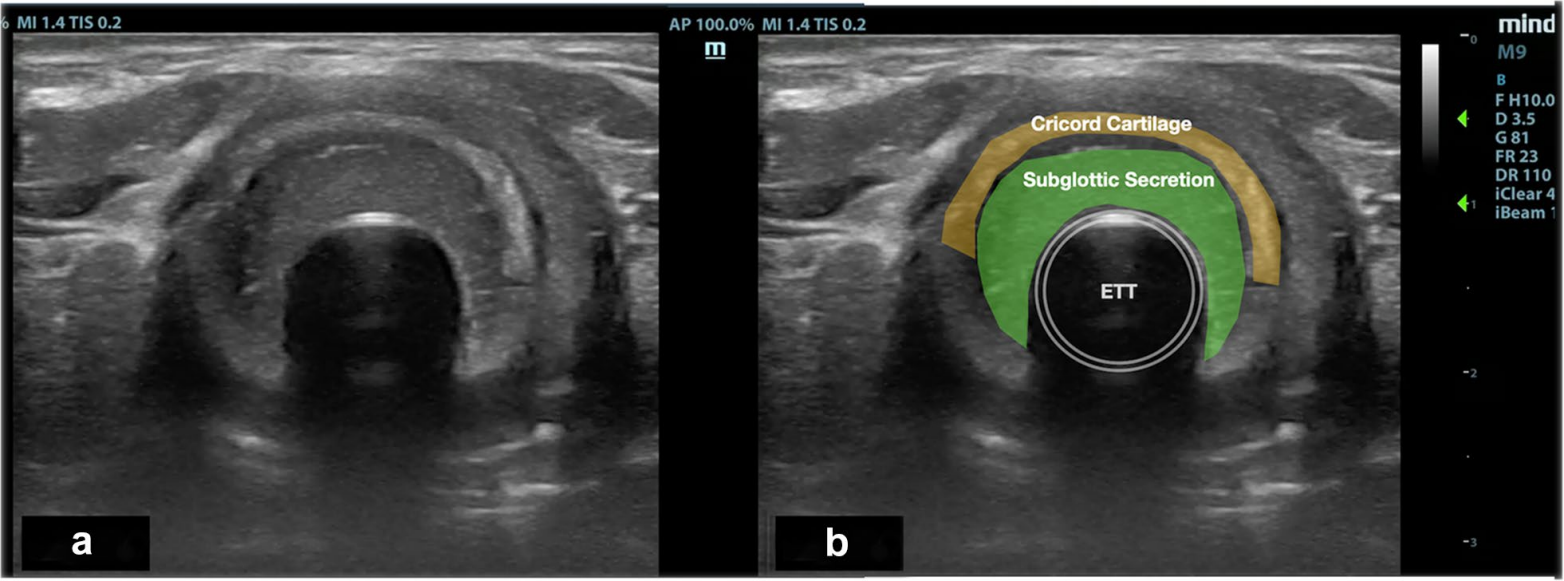
**a** A transverse view was obtained with the transducer placed across the anterior surface of the neck at the level of cricoid cartilage. **b** A cross-section of the cricoid cartilage (yellow watermark), ETT (white line) and presence of subglottic secretions (green watermark) were depicted

**Fig. 3:**
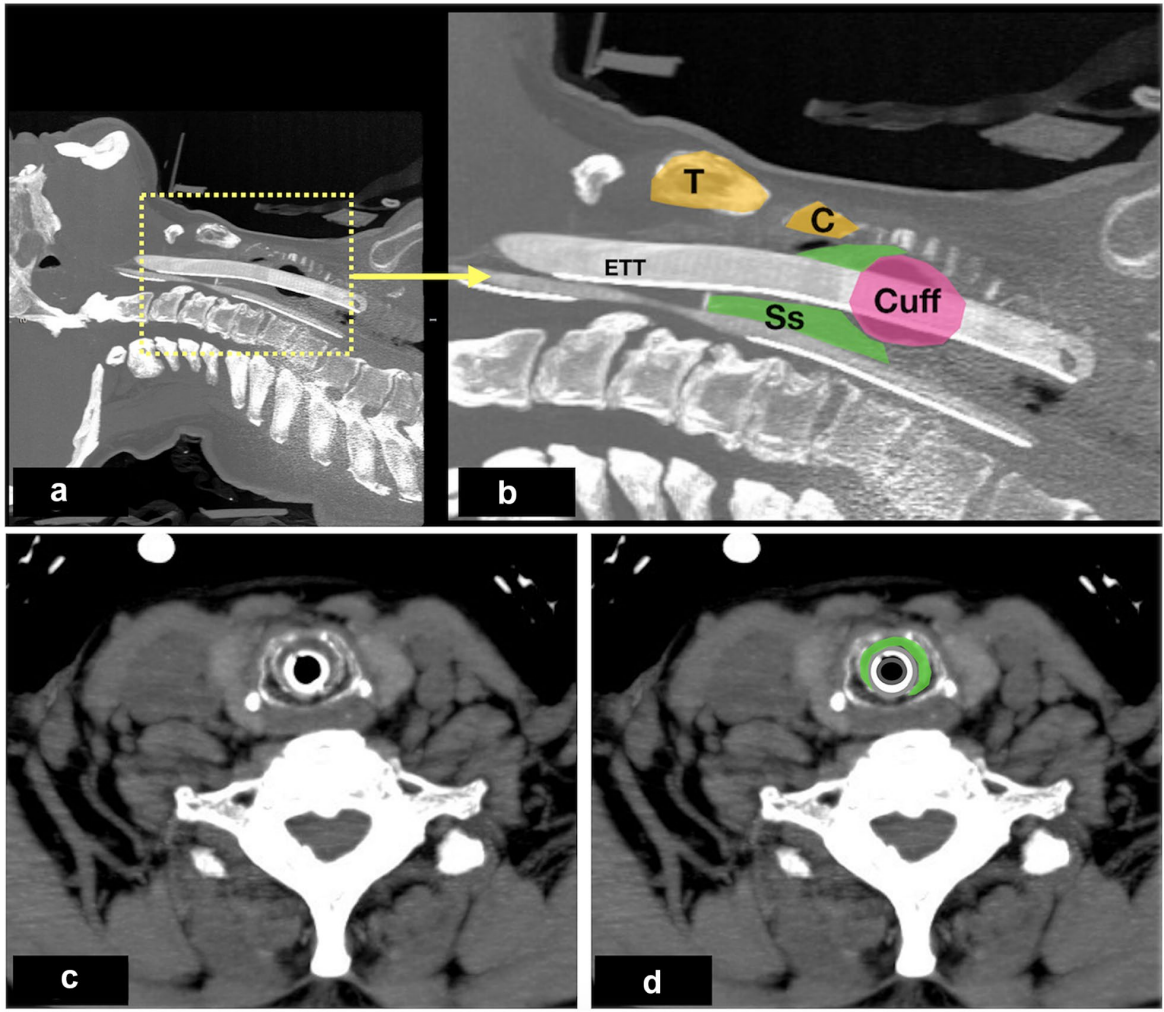
**a** and **b** A sagittal view of cervical CT scan showed a subglottic secretions (green watermark) above the ETT cuff (pink watermark) and its relation with other structures; **c** and **d** axial views of cervical CT showed subglottic secretions. *Ss* subglottic secretion; *C* cricoid cartilage; *T* thyroid cartilage; *ETT* endotracheal tube

Airway US was performed with the Mindray M9 US machine, using a 7.5 MHz linear array probe (Mindray M9, UMT-500 Plus, Germany, 2016). A standard scanning protocol was used (Figs. 1, 2 and 3).

A sagittal (longitudinal) view examination was performed at anterior midline of the neck to identify the air–mucosal (A–M) interface, ETT cuff, surrounding structures of importance such as the thyroid cartilage, cricoid cartilage, cricothyroid membrane and tracheal rings (Fig. 1, Additional file 1: Video S1). Transverse view examination was performed at the level of cricoid cartilage transversely across the anterior surface of the neck (Fig. 2, Additional file 2: Video 2). In order to acquire the image of subglottic secretion at the posterior part of ETT cuff, a parasagittal (lateral to the midline) scan was performed at the lateral right side of the neck with the transducer tilted towards caudad (Additional file 1: Video S3). The presence of subglottic secretions was defined as heterogenous or homogenous fluid collections or comet-tail artefacts caused by bubble-rich secretions above the ETT cuff [10].

The air–mucosal interface was observed as bright hyperechoic mobile lines. The thyroid and cricoid cartilages both had oval hypoechoic appearance in the parasagittal view and appeared like a hump in the transverse view. The thyroid cartilage was more anterior and larger in size compare to the cricoid cartilage. In longitudinal plane, the tracheal cartilage was seen as a “string of beads” and inverted U in the transverse plane. A failed US consisted in the inability to identify key anatomical structures or the inability to visualize the ETT balloon cuff [13, 14].

Neck CT scan was performed using a 64-multislice detector CT machine (Toshiba Aquilion CX 2010, Japan). CT findings were examined by a radiologist with more than 10 years of experience. The investigator radiologist and US operators were blinded to the findings obtained with the other technique.

### Sample size considerations

We calculated the sample size to determine whether an area under the curve (AUC) of ≥ 0.75 was achieved for a receiver operator characteristic (ROC) plot of neck US for detecting subglottic secretions versus cervical CT as a gold standard. The null hypothesis was set as AUC 0.5 (meaning no discriminating power), Type 1 error of 0.05 and power of 80%. Based on the unpublished data from our own experience, with a precision of 10%, and an expected proportion of subglottic secretions on chest CT scan of 80%, the sample size required was 45. Taking into account the potential for 10% incomplete data from neck US or cervical CT, we included 49 patients for the final analysis. AUROC sample size calculation was performed using MedCalc for Windows, version 19.4 (MedCalc Software, Ostend, Belgium). [15]

### Statistical analysis

The characteristics of the patients were summarized as medians and interquartile ranges for continuous variables, and as numbers and percentages for qualitative variables. The ROC curve and AUC estimates were determined for the relationship of neck US and cervical CT to diagnose subglottic secretions. Sensitivity (Se), specificity (Sp), negative predictive value (NPV) and positive predictive value (PPV) were provided with their 95% confidence intervals (CIs).

ROC and AUC, Se, Sp, NPV and PPV were determined for neck US to diagnose subglottic secretions according to the cervical CT findings as a gold standard. For all calculations, SPSS Statistics for Windows, Version 20.0 (IBM, Armonk, NY, USA) were used. The significance level was set at *p* < 0.05.

The inter-observer and intra-observer agreement percentages were calculated by dividing the number of occasions of agreement by the total number of occasions. Weighted kappa statistics were applied to determine the degree of agreement. The kappa statistics was interpreted as follows: less than 0.00, poor agreement, 0.00–0.20, slight agreement; 0.21–0.40, fair agreement; 0.41–0.60, moderate agreement; 0.61–0.80, substantial agreement and 0.81–1.00, almost perfect agreement. The level of statistically significant difference was *p < 0.01*. Statistical analyses were performed with SAS software version 9.1 (SAS Institute) [16].

## Results

### Characteristic of the patients

**Fig. 4:**
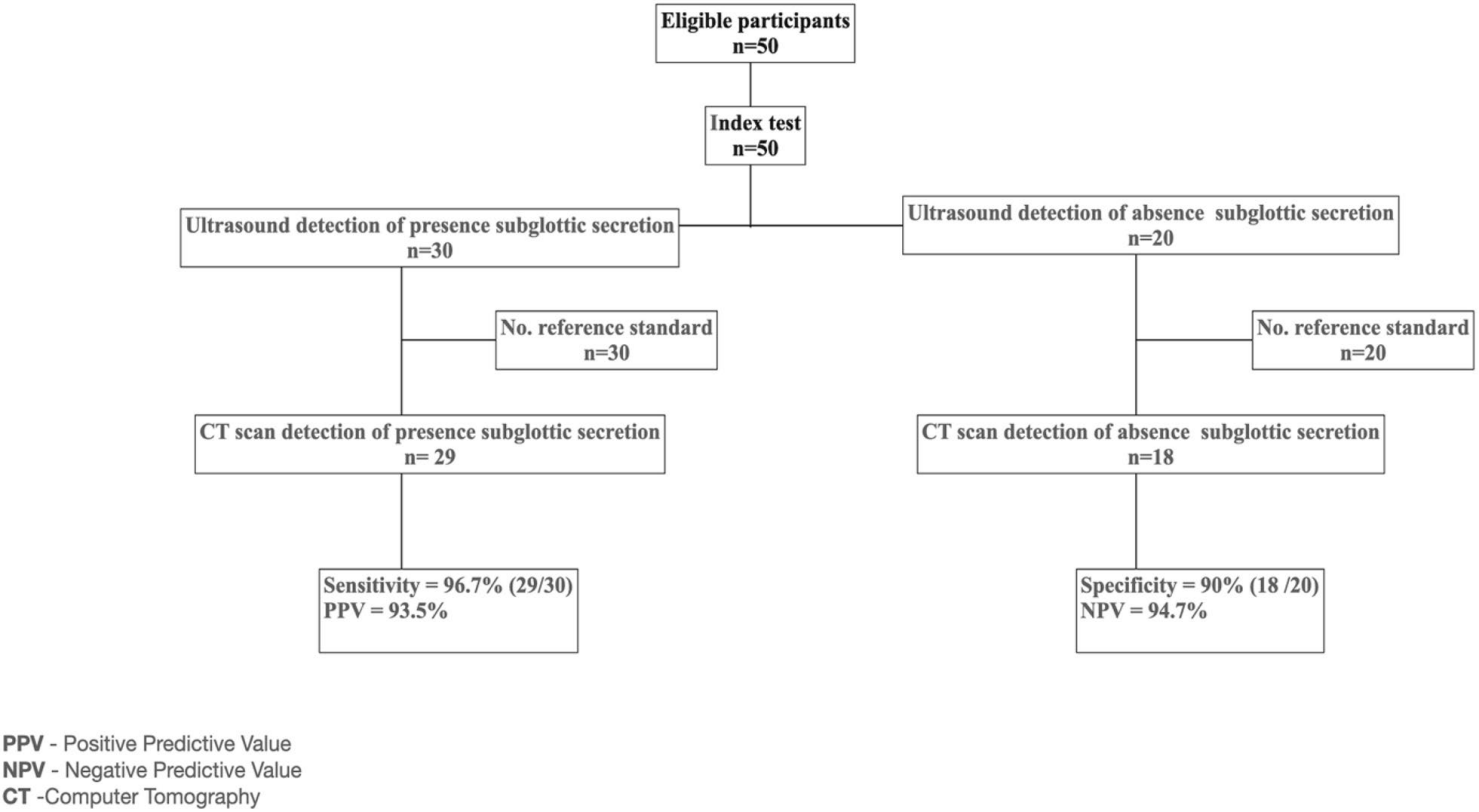
STARD flow diagram

During the study period, 50 intubated adult trauma patients (39 men and 11 women with a mean age 42 years old were recruited (Fig. 4).The indication for intubation were severe head injury in 42/50 (84%), chest injury in 6/50 (6%), cardiac and vascular injury in 2/50 (4%), cervical injury in 2/50 (4%), and abdominal injury in 1/50 (2%). The mean time from intubation to bedside airway US was 3.7 h (± 2.5 h). There was no significant difference between patients with subglottic secretions and those with no subglottic secretions in terms of comorbidities, ETT size, Glasgow Coma Scale on arrival, Sequential Organ Failure Assessment score, Acute Physiology and Chronic Health disease Classification System II (APACHE II) score, Simplified Acute Physiology Score (SAPS II) score and antibiotic use (Table 1).

**Table 1:** Baseline characteristic

	Subglottic secretions present *n* = 31	Subglottic secretions absent *n* = 19	*P* value
Gender, male, n (%)	23 (74)	16 (84)	0.407^a^
Female, n (%)	8 (26)	3 (16)	
Age, mean (95% CI), year	38 (33.6–42.9)	49 (42–56)	0.01^b^
Subglottic secretion score, n (%)	0 (absent) = 19 (38)		
	1 (posterior only) = 6 (12)		
	2 (anterior and posterior) = 25 (50)		
Comorbidities			0.231^a^
None, n (%)	25 (81)	15 (79)	
Multiple,n (%)	3 (10)	4 (21)	
Diabetes mellitus, n (%)	3 (10)	0 (0)	
Medical treatment given			
Antibiotic usage, n (%)	6 (19)	3 (16)	0.532^a^
Physiological parameter			
Respiratory rate, mean (95% CI)	20 (19–21)	21 (19–23)	0.533^b^
Mean arterial pressure, mean (95% CI)	97 (91–102)	91 (85–98)	0.217^b^
Heart rate, mean (95% CI)	94 (87–91)	94 (82–107)	0.931^b^
SpO_2_, mean (95% CI)	99 (99–100)	96 (94–99)	0.006^b^
ETT size, mean (95% CI)	7.7 (7.6–7.8)	7.8(7.6–8.0)	0.356^b^
GCS, mean (95% CI)	6.3 (5.2–7.4)	7.6 (6.0–9.2)	0.144^b^
SOFA score mean (95% CI)	4.5 (3.6–5.3)	4.0 (3.1–4.8)	0.426^b^
APACHE score, mean (95% CI)	13.5 (11.1–15.9)	13.9 (11.5–16.3)	0.816^b^
SAPS score, mean (95% CI)	31.2 (27.5–34.9)	31.8 (26.8–36.7)	0.846^b^
Outcomes			
VAP, n (%)	18 (58)	2 (11)	0.01^a^
Mortality, n (%)	6 (19)	6 (32)	0.258^a^
ICU stay (days)	4.7 (2.2–7.1)	9.4 (3–15.8)	0.099^b^
Hospital stay (days)	8.5 (5.7–11.2)	14.8 (6.9–22.7)	0.069^b^
Ventilator usage (days)	4.0 (1.9–6.1)	9.2 (2.7–15.6)	0.062^b^
SOFA at discharge, mean (95% CI)	2.4 (0.7–4.1)	3.5 (1.5–5.4)	0.404^b^

^a^Chi-square

^b^*t-*tes*t*

### Primary outcome

Airway US had a sensitivity of 96.7% (29/30) and specificity of 90% (18/20) in diagnosing subglottic secretions. The negative predictive value (NPV) was 94.7% and positive predictive value (PPV) was 93.5% (Fig. 4).

### Secondary outcomes

There was significantly higher incidence of VAP in patients with subglottic secretions compared to those with no subglottic secretions (58% vs 11%, *p = 0.01*). However, there was no significant difference between the groups in terms of mortality, ICU length of stay, hospital length of stay, and days on ventilator (Table 1).

### Inter-rater reliability

**Fig. 5:**
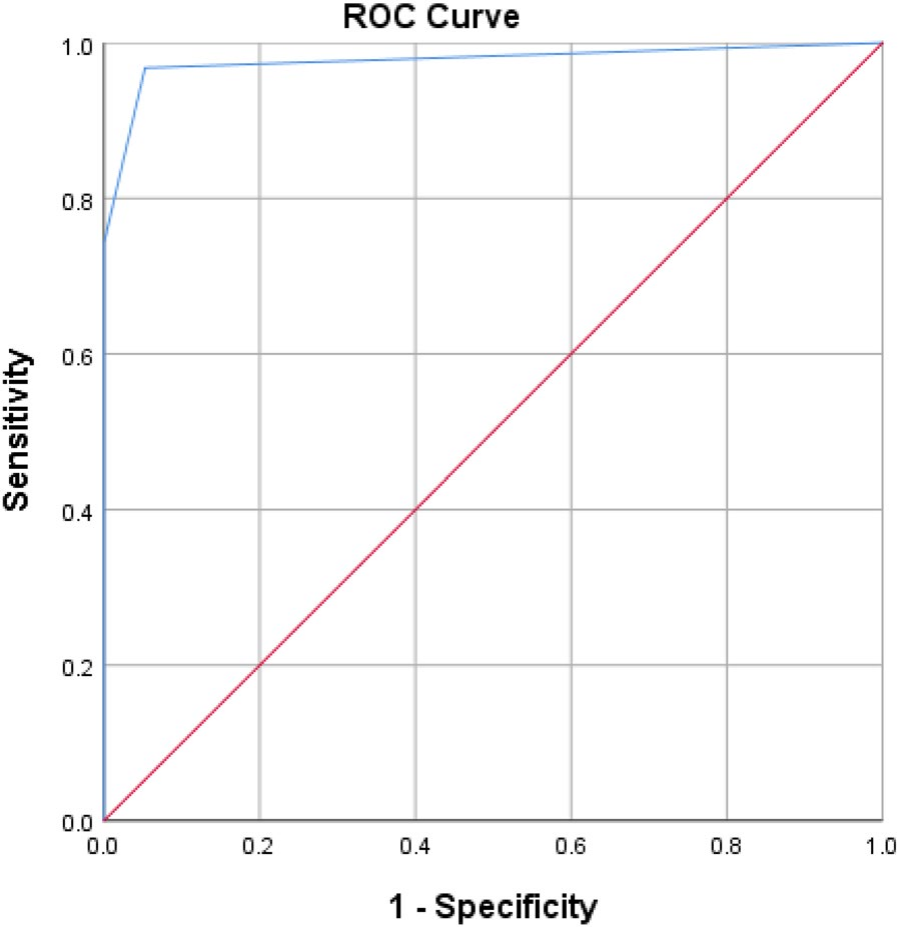
Area under receiver operating curve (AUROC) US Subglottic Secretion Score vs gold standard (CT cervical detection of subglottic secretion) AUROC = 0.977, *p < 0.001*

The inter-rater reliability between three emergency physicians calculated by Fleiss Multirater Kappa was 0.923 (95% CI 0.916, 0.930) in 24 patients (Fig. 5).

## Discussion

Proper management of either bronchial secretions or subglottic secretions is of utmost importance to prevent VAP in intubated patients [5], in addition to maintaining the ETT cuff pressure within the recommended target values [17]. VAP incidence is lower in patients who have been intubated with specialized ETT that included a subglottic secretion drainage port [17]. While the impact of subglottic secretion aspiration are often recognized when VAP occurs, airway US, as shown in our study, may be able to detect subglottic secretions repeatedly at the patient’s bedside before aspiration occurs, aiding in providing a timely subglottic drainage and preventing microaspiration. In addition, airway US may aid in determining positioning of the ETT cuff and adjust ETT depth as needed [14].

Compared with US, advanced techniques such as CT [18] or magnetic resonance imaging delineate with precision the subglottic space [19]. However, the main indications include detecting of subglottic stenosis, laryngeal tumours and neck trauma [19, 20]. Furthermore, these modalities require moving the patient to the radiology department, are costly, and expose the patient to ionizing radiation (CT). In contrast, point-of-care US is widely available in the emergency department or ICU, is cheaper, does not expose the patient to ionizing radiation, and can be performed at the patient’s bedside. US has been proven to assist in detecting endotracheal vs. esophageal intubation, with a short learning curve. In addition, it provides adequate images of the subglottic space to detect subglottic secretions and the position of the ETT cuff [21–24].

The detection of subglottic secretions using plain radiograph had been described by Greene et al. in 1994 [8]. Using airway US to observe at the subglottic area is recent. Tao et al. first demonstrated ultrasound-guided visualization of subglottic secretions in an intubated patient, by injecting saline through the subglottic catheter above the ETT cuff [10]. This was followed by a case report by Yan et al., who detected subglottic secretions in a patient who had gastric regurgitation while undergoing general anaesthesia [11].

Our study showed that airway US can be a reliable tool to visualize subglottic secretions in intubated patients at the emergency department and their early visualization may lead to secretion aspiration and eventually reduce microaspiration. Continuous SSD had been found to be associated with trachea mucosa damages. Intermittent SSD when there are accumulation subglottic secretions detected by ultrasound may help to mitigate this risk [6, 7, 25]. However, this hypothesis should be tested in an appropriately design and powered trial also to evaluate prognostic prevention of VAP.

## Limitation

The limitation of our study is the small sample size, and the population selected that are exclusively trauma patients. There was also a selection bias as patients with airway trauma were excluded. However, our findings may be replicated to other types of patients.

An important limitation is the correlation with VAP since our study only observed for subglottic secretions at only one point in time (i.e. before patient admission to the ICU). The best frequency to perform US and subglottic suctioning has yet to be determined. Regular US detection of subglottic secretions synchronized with subglottic suctioning could more clearly define the impact on VAP and can be a matter of future studies.

## Conclusion

Upper airway US has excellent sensitivity and specificity for the detection of subglottic secretions in adult trauma patients. Further studies in non-trauma patients are needed to corroborate this findings.

## Potential usage of this knowledge

- Optimizing the frequency of subglottic suctioning.
- Demonstrating the correlation between subglottic secretion detection on airway US and VAP incidence.
- Optimize the depth of the ETT.

## Supplementary Information

**Additional file 1: Video S1.** A longitudinal view was performed at the anterior midline of the neck to identify the air–mucosalinterface, endotracheal tubecuff, and surrounding structures of importance such as thyroid cartilage, cricoid cartilage, cricothyroid membrane and tracheal rings.

**Additional file 2: Video S2.** A transverse view was performed across the anterior surface of the neck at the level of cricoid cartilage.

**Additional file 3: Video S3.** A transverse view was performed across the anterior surface of the neck at the level of cricoid cartilage.

## Data Availability

The materials are available from the corresponding author on reasonable request.
